# Supplementary material for: Resistance to BRAF Inhibitors: EZH2 and Its Downstream Targets as Potential Therapeutic Options in Melanoma
Source: Int J Mol Sci. 2023 Jan 19;24(3):1963. doi: 10.3390/ijms24031963 (PMC9916477; doi:10.3390/ijms24031963)
Supplement: Supplementary file 1 [file ijms-24-01963-s001.zip › ijms-2103792-supplementary.pdf]

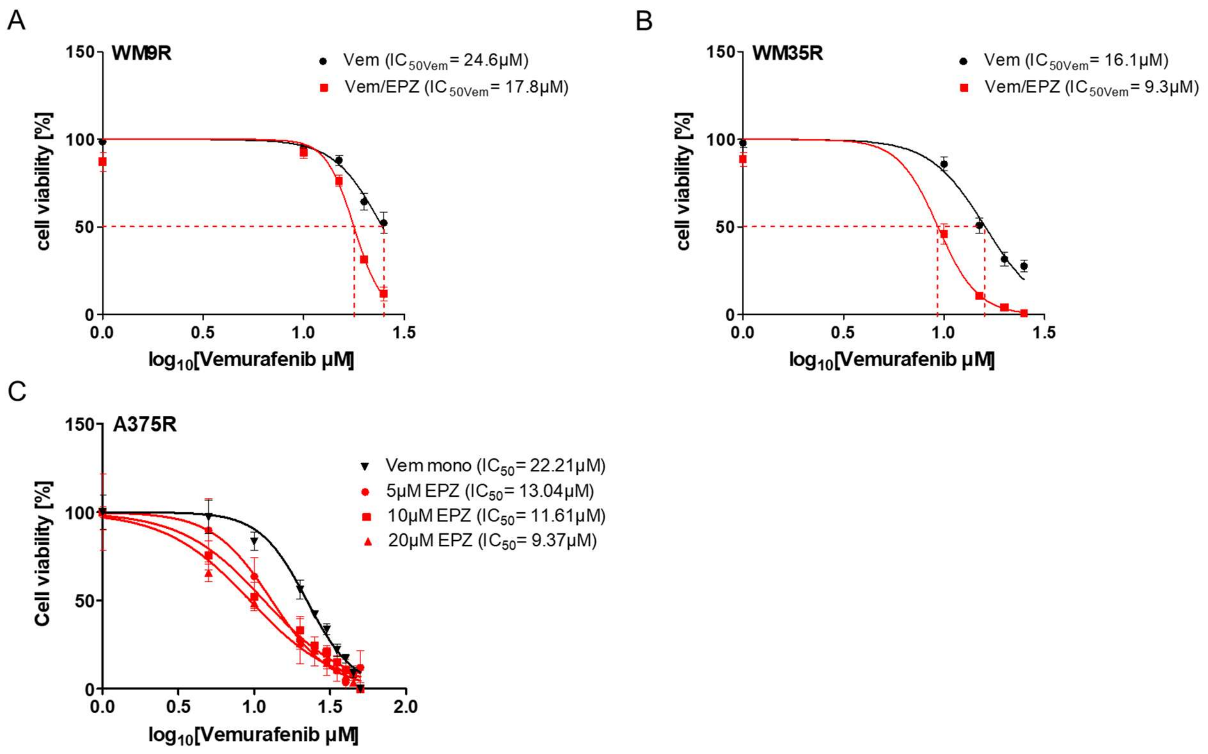

Supplemental Figure S1. Inhibition of EZH2 improves response to Vemurafenib in different resistant melanoma cell lines. Viability analyses of Vemurafenib (VEM) resistant cell lines **A)** WM9R and **B)** WM35R treated with increasing concentrations of Vemurafenib monotherapy or in combination with EPZ (5 µM). **C)** Cell viability assay of Vemurafenib resistant A375R cells with increasing concentrations of Vemurafenib monotherapy or in combination with different EPZ concentrations (5 - 20 µM).

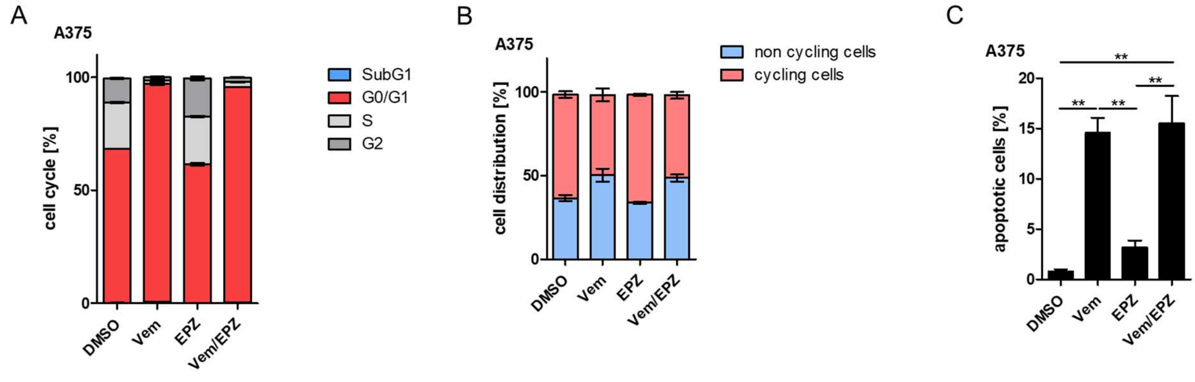

Supplemental Figure S2. Combination of Vemurafenib and EPZ has no additional effect compared with Vemurafenib alone on A375 cells. **A)** Cell cycle analyses of Vemurafenib susceptible A375 cells treated with DMSO, Vemurafenib (Vem) (1 µM), EPZ (5 µM) or Vemurafenib and EPZ in combination for 72 h. **B)** Bar chart represent cycling and non-cycling A375 cells after serum starvation and treatment as described above. **C)** Analysis of apoptosis for A375 cells treated as indicated. (** $p < 0.01$)

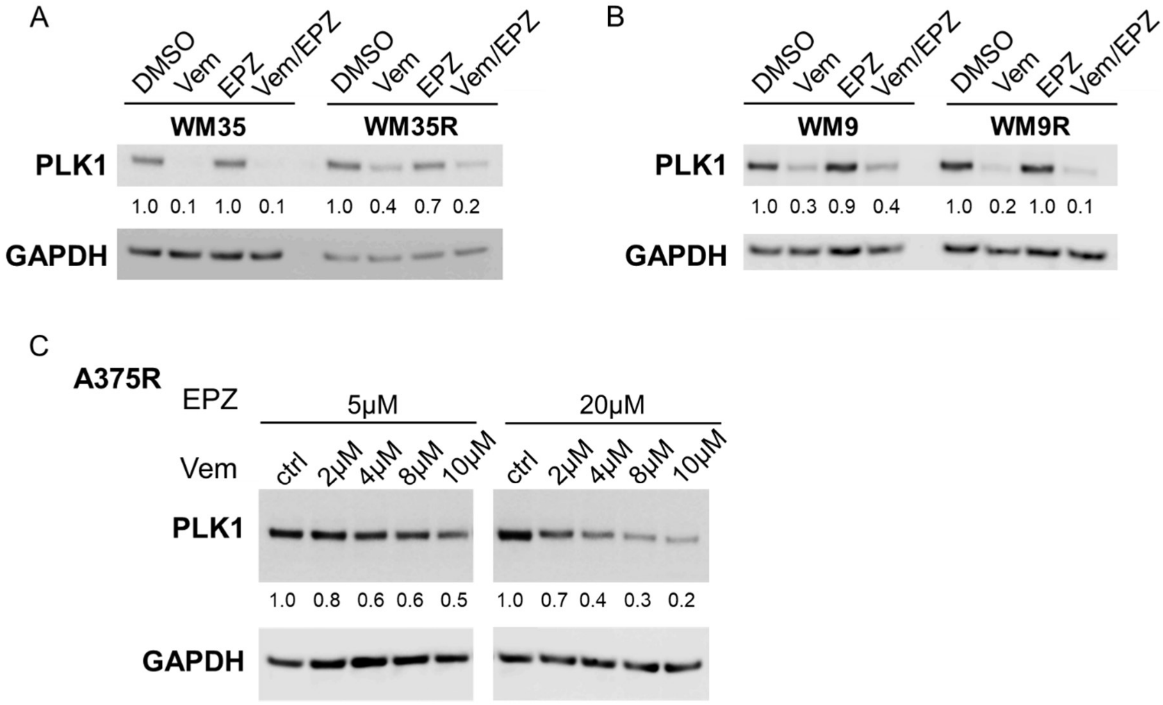

Supplemental Figure S3. Vemurafenib treatment decrased PLK1 protein expression. Western blot analysis of **A)** WM35 and WM35R as well as **B)** WM9 and WM9R, treated with DMSO, Vemurafenib (Vem) (susceptible cells 1 µM and resistant cells 10 µM), EPZ (5 µM) or Vemurafenib and EPZ, examined PLK1 and GAPDH. **C)** Western blot of A375R cells, treated with increasing concentrations of Vemurafenib (0 – 10 µM) in combination with two different concentrations of EPZ (5 and 20 µM), investigated PLK1 and GAPDH.

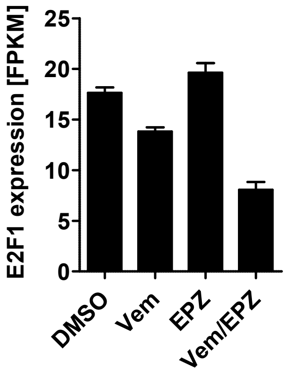

Supplemental Figure S4. E2F1 expression is downregulated after treatment with Vemurafenib or Vemurafenib and EPZ. E2F Transcription Factor 1 (E2F1) expression (fragments per kilobase of transcript) in A375R cells treated with DMSO (control), EPZ (5 µM), Vemurafenib (Vem) (10 µM) or Vemurafenib and EPZ.
